# Diagnosing COPD in primary care: what has real life practice got to do with guidelines?

**DOI:** 10.1186/s40248-019-0191-6

**Published:** 2019-09-09

**Authors:** Greta Ragaišienė, Rūta Kibarskytė, Rasa Gauronskaitė, Monika Giedraitytė, Agnė Dapšauskaitė, Vytautas Kasiulevičius, Edvardas Danila

**Affiliations:** 1 Clinic of Internal Diseases, Family Medicine and Oncology of Vilnius University, Santariškių st. 2, Vilnius, Lithuania; 2 Center of Family Medicine of Vilnius University Hospital Santaros Klinikos, Taikos st, 104-52, Vilnius, Lithuania; 3 Clinic of Chest Diseases and Allergology of Vilnius University, Vilnius, Lithuania; 4 Center of Pulmonology and Allergology of Vilnius University Hospital Santaros Klinikos, Vilnius, Lithuania; 5 Faculty of Medicine, Vilnius University, M. K. Čiurlionio st. 21/27, Vilnius, Lithuania

**Keywords:** Chronic obstructive pulmonary disease, COPD in primary care, COPD guidelines, Spirometry, COPD grading

## Abstract

**Background:**

The role of primary care physician in COPD management varies in different health care systems. According to the researches in various countries, extent of spirometry use in diagnosing and grading COPD frequently remains insufficient. Inaccurate diagnosis results in mistreatment and disease progression.

The aims of our study were to investigate the accuracy of COPD diagnosis, grading, and treatment according to guidelines in daily practice of primary care.

**Methods:**

A retrospective analysis of ambulatory records in a large primary care center was conducted. Digital medical records of current patients were screened for ICD-10-AM codes J44.0, J44.1, J44.8 and J44.9. All medical records starting from the first visit in this primary care center were reviewed.

**Results:**

Two hundred twenty-eight patients diagnosed with COPD were included in the study, 118 male, mean age 67 yrs. (SD 14). A spirometry report was available to 58% of the patients, 75% of them met the guidelines for COPD diagnosis. The grade was correct for 56.8% of the patients. 54% were consulted by the pulmonologist at least once. After re-analyzing spirometry, correcting the diagnosis, and grading, it was determined that only 70% of the patients were receiving appropriate treatments. Sixteen per cent of patients were undertreated and 14% were overtreated.

**Conclusions:**

COPD care in primary practice remains suboptimal. Incorrect approach often leads to incorrect grading and mistreatment. Points for improvement should be identified in further studies.

## Background

Chronic obstructive pulmonary disease (COPD) is a common, preventable, and treatable illness, characterized by persistent respiratory symptoms and airflow limitation due to airway and/or alveolar abnormalities usually caused by significant exposure to noxious particles or gases [1]. According to the World Health Organization, it is the third most common cause of death in the world [2]. Late and inaccurate diagnosis results in mistreatment and disease progression causing the treatment costs to double, mainly due to more frequent exacerbations [3].

The role of the primary care physician in COPD management varies in different health care systems. As an example, in the US primary care physicians are responsible for diagnosing, managing, and coordinating care of the COPD patients [4]. In European countries the role of the primary care physician varies from diagnosing, managing COPD, and taking the role of care coordinator to minimal involvement as COPD care is mainly led by pulmonologists [5, 6]. In Lithuania primary care physicians are responsible for identifying early signs of COPD and referring the patients to a pulmonologist for diagnosis, grading, and treatment planning. After COPD treatment is prescribed, the family physician continues long term observation and management. According to local guidelines [7] and legal documents, [8] COPD diagnosis is based on FEV_1_/FVC post-bronchodilator ratio and the grading of airway obstruction is based on FEV_1_, as recommended by the GOLD guidelines [1].

The aim of our study was to investigate accuracy of COPD diagnosis, grading, and treatment according to local recommendations in daily practice in primary care.

We conducted our study in a large primary care center which is a department of a tertiary referral center located in Vilnius, the capital of Lithuania. The center has 12 primary care physicians and provides care for approximately 10,000 patients per year. It also has excellent availability of various specialist consultations, laboratory, imaging, and pulmonary function testing. The center is fully equipped with an electronic medical record system.

## Methods

A retrospective cross-sectional analysis of ambulatory records in the primary care center was conducted. Data were collected from January 2017 to October 2017. Digital medical records of all current patients were screened for ICD-10-AM codes J44.0, J44.1, J44.8 and J44.9. All digital and handwritten medical records starting from the patients’ first visit in this primary care center were reviewed. Patients registered with previously mentioned ICD-10-AM codes were included in the study. Data about spirometry results, radiological imaging and comorbid disease, previous pulmonologists consultations were collected.

Spirometry was performed in the department of Pulmonology and Allergology of University Hospital where approximately 9,000 various pulmonary function tests are being performed annually. All testing was supervised by a specialized nurse and performed according to the guidelines of the American Thoracic Society/European Respiratory Society. The parameters were measured using Vmax Encore (Viasys® Healthcare, US). Reversibility testing was performed if obstruction was present. The evaluation of the spirometry was conducted by a dedicated pulmonologist. Records of spirometry results are kept in ambulatory cards and electronic medical record system. To confirm or deny the diagnosis of COPD and define the bronchial obstruction severity, all available patient spirometry reports were analyzed. Congruence of the diagnosis, grading and treatment of the disease to local guideline recommendations corresponding to GOLD guidelines was evaluated.

Statistical analysis was performed by using SPSS 17.0 software package. *P*-values were two-tailed with a value < 0.05 considered to be statistically significant. Continuous and normally distributed variables are presented as mean ± standard deviation (SD). Normality assessment was done by using visual inspection, skewness and kurtosis measures, and Kolmogorov-Smirnov test. Categorical data are presented as counts and percentages. One-way ANOVA for parametric data and Kruskal-Wallis test for nonparametric data comparisons were used.

## Results

### Population characteristics

228 patients diagnosed with COPD were included in the study. The demographic data are presented in Table 1.

**Table 1:** Demographic data

	All patients (*n* = 228)	COPD confirmed by spirometry (*n* = 99)	COPD unconfirmed by spirometry (*n* = 33)	No spirometry data available (*n* = 96)	*P*
Age, yrs.	67 (±14)	70 (±13)	66 (±12)	64 (±16)	<0,05
Male, %	51.8	59.60	57,60	41.70	<0.05
Female, %	48.20	40.40	42,40	58.30	<0.05
Current smoker, %	21.10	28.30	39.40	7.30	<0.05
Smoking status not recorded, %	51.30	23.20	30.30	87.50	<0.05
Living in a rural area, %	19.70	23.20	27,30	13.50	>0.05
Mean comorbidities, n (±SD)	1.5 (±1.7)	2.5 (±1.5)	2.3 (±1.6)	0.5 (±0.8)	<0.05

### The accuracy of diagnosing and grading COPD

**Fig. 1:**
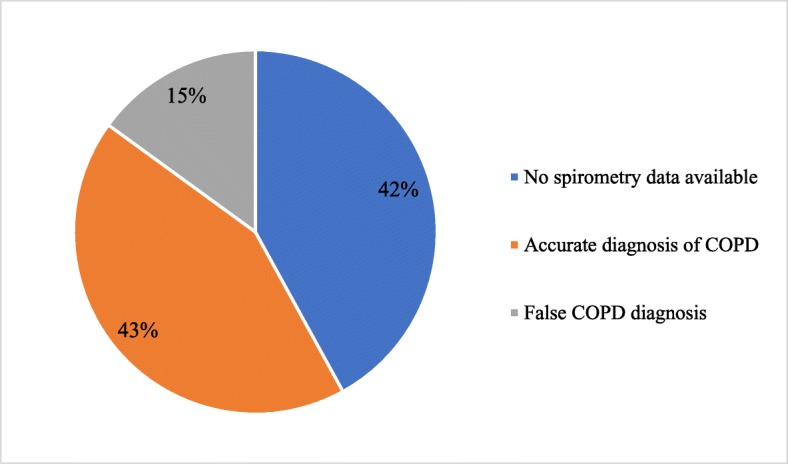
Accuracy of COPD diagnosis

A spirometry report was available only to 58% of the patients. Only 99 (75%) of them met the guidelines for COPD diagnosis. The information about spirometry availability and COPD diagnosis accuracy according to spirometry is presented in Fig. 1.

In the group of 132 patients whose spirometry results were available, the grade was stated for 95 (72%) patients. However, the grade of the disease was correct only for 54 (56.8%) patients. For 24 (25.3%) patients the grade was determined incorrectly, for 9 (9.5%) of them the grade was underestimated, 15 (15.8%) overestimated. 17 (17.9%) patients were diagnosed with COPD and the grade of the disease was specified even though their spirometry results did not meet the GOLD criteria for diagnosis.

**Fig. 2:**
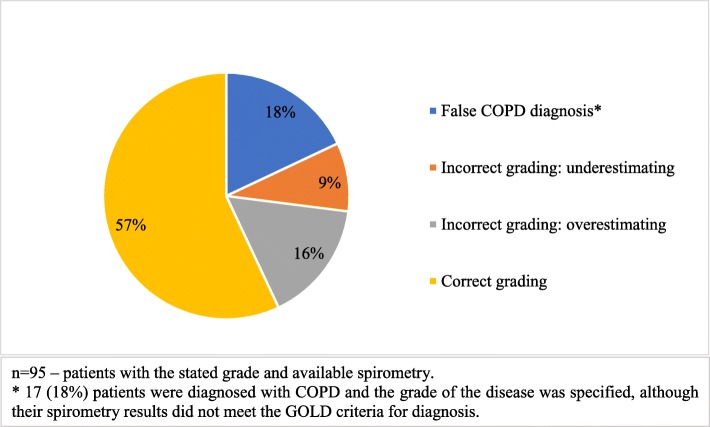
Accuracy of diagnosis formulation and grading

6 (4.5%) patients had a COPD diagnosis written down but did not have a grade determined, 5 of these patients did not meet the diagnostic criteria for COPD. 31 (23.5%) patients had no grade of COPD determined and had a handwritten diagnosis of asthma or chronic bronchitis, although 20 (15.2%) of them met the COPD diagnostic criteria. The grading accuracy is demonstrated in Fig. 2 (Accuracy of diagnosis formulation and grading). The distribution of patients according to COPD grade after spirometry reevaluation is presented in Table 2.

**Table 2:** COPD grade after spirometry reevaluation

COPD stage	Patients, n (%)
I	51 (38.6)
II	39 (29.5)
III	9 (6.8)
IV	0 (0)
False diagnosis	33 (25.0)

### Pulmonologist consultation and further testing

Out of all patients, only 54% (122) were consulted by the pulmonologist at least once, although, according to the local guidelines, COPD diagnosis must be confirmed by the pulmonologist. Out of the 122 pulmonologists’ consulted patients, 118 had spirometry results available and 92 of them had COPD according to the diagnostic criteria. For 41 (45%) of these patients the grade of COPD was set incorrectly. However, we cannot determine whether the diagnosis was made incorrectly by the pulmonologist, or if the primary care physician overlooked or ignored the pulmonologist’s adjustments.

### COPD treatment according to grading

Only 93 patients’ records had enough information about the treatment for evaluation. Majority of these patients (80%; 74) were prescribed with the treatment as recommended by the local guidelines. After re-analyzing spirometry, correcting the diagnosis, and grading, it was determined that only 70% (65) of the patients were receiving appropriate treatments. 16% (15) patients were undertreated, 14% (13) were overtreated.

**Fig. 3:**
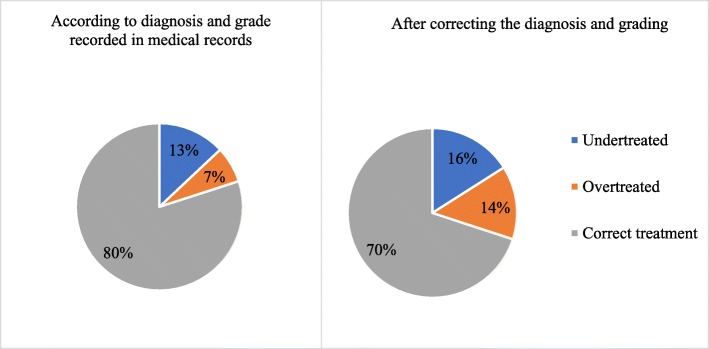
Accuracy of treatment

The study data indicated various treatment contradictions to local guidelines: 8% (8) of patients with mild COPD were treated with a long-acting β_2_ agonist and inhaled corticosteroid combination therapy, 10 (10%) patients who had moderate or severe COPD were not prescribed with a long acting inhaled bronchodilator, 4 (5%) patients who had moderate or severe COPD received no treatment at all. The comparison of treatment accuracy according to original COPD grade and grade set after reevaluation is presented in Fig. 3.

## Discussion

This study reports the accuracy of diagnosis, grading, and treatment of COPD according to guidelines in daily practice. Despite the wide availability of local COPD guidelines [7], corresponding to international guidelines, this study suggests that primary care physicians’ clinical practice is often not in compliance with the current recommendations.

Fifty-eight percent of the patients were not referred for spirometry even though the necessity of spirometry in diagnosing, grading and treating COPD is well known. Research in various countries demonstrates that the extent of spirometry use in diagnosing COPD and grading frequently remains insufficient. Two studies conducted in the U.S. in the 2000s stated that only a third of the patients with newly diagnosed COPD had undergone a spirometry [9, 10]. Varying results are seen in Europe, from 30% in Italy and Spain, to 59% in Sweden [11–14].

Our study shows that despite the constant education efforts in Lithuania, recognizing and diagnosing COPD still remains a challenge. According to current data, spirometry use was reported for nearly half of the patients. These results look respectable in the context of the previously presented data. However, this might be due to the characteristics of the chosen primary care center as it is a large, well-equipped center situated in the capital city with convenient learning opportunities for family physicians. The aforementioned center provides an easy access to spirometry, other diagnostic tests, and consultants. It is possible that the situation might be worse in rural areas where various health care aspects are out of reach.

Spirometry continues to be underused despite its accessibility. As studies show [15], the availability of spirometry is not viewed as a barrier. The main reason for underuse of spirometry might be the lack of knowledge or resources which leads to difficulties in performing or interpreting the results. Even more importantly, these factors might contribute to cultivating a misconception that spirometry is not needed to diagnose COPD [15].

Study [16] suggests that the lack of recent training is not the main reason of insufficient use of spirometry. Lack of resources, such as insufficient time, complicated logistics and high expenses, further complicate adhering to guidelines. For example, in Lithuania the usual primary physicians’ consultation time for one patient is 10 to 20 min, excluding non-scheduled visits. The average consultation time of the primary care physician was reported to be 15 min and was shown to vary from 48 s in Bangladesh to 22.5 min in Sweden [17]. Providers who felt limited by time and felt unable to integrate onsite spirometry into stream of patients were less likely to use spirometry to diagnose or assess COPD [18, 19].

Our study demonstrated that even if spirometry was performed, primary care physicians diagnosed COPD in 15% of patients that did not meet the spirometry diagnostic criteria. Other studies show variable degrees of misdiagnosis: from 10% [20, 21] up to over 40% of patients [22, 23]. Comparison of COPD diagnosis made in primary care against the diagnosis made in secondary care shows that primary care specialists who are not specialized in diagnosing COPD make mistakes more often than experts. In primary care, misdiagnosis of COPD may be attributed to the lack of awareness and knowledge of the disease which leads to the poor use of spirometry, as mentioned before [9–14]. Without the appropriate knowledge of the guidelines, physicians rely on clinical signs and anamnesis when diagnosing COPD.

Our study showed that primary care physicians confuse COPD with asthma and chronic bronchitis. In a multicenter US study approximately 40% of the patients who were previously diagnosed with asthma had COPD [24]. This might be associated with the lack of knowledge in pulmonary diseases in general.

Multimorbidity is another factor of misdiagnosing COPD. Studies show that 38% of the heart failure patients are misclassified as having COPD [25]. Furthermore, 25% of the patients with ischemic heart disease met the COPD criteria. Eighty-two percent of these patients were underdiagnosed [26]. It seems that cardiovascular pathology can lead to both over and underdiagnosis of COPD. Although in literature comorbidities are associated with diagnostic inaccuracy, [27] in our study the number of comorbidities in patients with spirometry confirmed COPD and the spirometry unconfirmed group did not differ significantly.

In our study a quarter of patients’ grading was incorrect. In majority of the cases the mistake was the overestimating of the grade. Similar results are seen elsewhere. In 2014 a UK study compared the grading of airway obstruction in the primary and specialist care conditions. The proportion of patients whose grade of airway obstruction was misclassified, was significantly greater in primary care (43.4%) than specialist care (9.3%). The grade of airway obstruction found on spirometric assessment at specialist respiratory nurse-led clinic was different in 54.0% from that stated on the referral from primary practice [20]. Inappropriate spirometry interpretation is one of the reasons for grade overestimating. Another reason might be that physicians evaluate pre-bronchodilator FEV_1_ instead of post-bronchodilator. A study on accuracy of diagnostic registers demonstrated that the severity grading of airflow obstruction based on pre-bronchodilator readings changed after bronchodilator in 18% of patients [28]. One more potential reason for grade misclassification might be the confusion between obstruction severity grading and disease burden severity. Even in light of the new recommendations to tailor the treatment to the individual needs of the patient, the obstruction severity has to stay one of the factors to be accounted for among symptoms and exacerbation rate [1].

Inhaled bronchodilators are the cornerstone of COPD management and can increase exercise capacity and improve health status when used regularly. However, data about the treatment in our study showed that 70% of the patients were receiving appropriate treatments.

In a large study performed in the United Kingdom, results were quite similar to ours [29]. The study demonstrated that for 59.8% patients with confirmed COPD inhaled medications were prescribed in line with 2009 GOLD guidelines. The overall guideline adherence to COPD treatment for population-based COPD cases has been reported significantly lower than amongst hospital-recruited COPD patients [30]. However, more patients appeared to be overtreated for their GOLD grade than in our study, 37.7% versus 8%, respectively (over-prescribing of inhaled corticosteroids in COPD has been reported in a number of countries [13, 28, 31–33]. The fact that the frequent mistake is the abuse of steroids or long acting β_2_ agonists may be explained with the hypothesis that primary care physicians often start therapy after a relapse and subsequently maintain the same therapy without further investigations. Likewise, underlying factor may be the perceived similarity of COPD and asthma, the common occurrence of the two diseases together, and a hope that steroids could reduce the impact of symptoms in COPD. Also, it has been observed that age and comorbidities seemed to guide prescriptions for COPD medications more often than the assessment of dyspnea [34].

The consumption of long-acting bronchodilators in patients who had moderate or severe COPD was high (90%) in our study. Unlike the results in the US survey on COPD, [35] which reported that only 35% of primary care physicians chose a long-acting bronchodilator when a short-acting agent had failed. A third of physicians also chose a combination short-acting bronchodilator. In general, it has been reported that the proportion of patients prescribed with long-acting β_2_ agonists ranges between 23 and 56% in different practices, [36] and between 9 and 25% for long-acting anticholinergics in different practices [28]. Surprisingly, even 4 (5%) patients who had moderate or severe COPD received no treatment at all.

### Limitations and strengths

We are aware of limitations and shortcomings of our research. The study was conducted in a single center so it might not reflect the real diversity of patients, their care tactics and care limitations. Nonetheless, chosen primary care center was expected to demonstrate best possible results as it has good availability of needed facilities. Another weakness of our study is a small sample size which could reduce its ability to detect smaller significant differences. However, all current COPD patients of the center were included so the results should represent the population adequately. Lack of patient history such as smoking or smoking cessation and information about patients’ complaints is another weakness. Due to retrospective design of the study, some influencing factors could not be evaluated as they were not documented.

## Conclusion

COPD care in primary practice remains suboptimal. Incorrect diagnostic approach often leads to incorrect grading and mistreatment of COPD. Effects of these inaccuracies on disease burden, exacerbations and mortality are unclear and should be assessed in further studies. Lengthening consultation time or appointing COPD centered follow up consultations could help clinicians to amend quality of care. Furthermore, additional evaluation by specialist or a trained in respiratory care nurse might decrease misguided evaluation of poorly performed spirometry and misdiagnosis and misclassification in general.

## Data Availability

The data that support the findings of this study are available from the corresponding author upon reasonable request.

## Abbreviations

COPD: Chronic obstructive pulmonary disease
FEV_1_: Forced expiratory volume in 1 s
FEV_1_/FVC: Ratio of FEV_1_ to FVC
FVC: Forced vital capacity
GOLD: Global Initiative for Chronic Obstructive Lung Disease
ICD-10-AM: International Statistical Classification of Diseases and Related Health Problems, Tenth Revision, Australian Modification
SD: Standard deviation
